# Neuropsychiatric Psychosis and Xanomeline in the treatment of an Adolescent with a MED13L mutation

**DOI:** 10.1192/j.eurpsy.2026.10420

**Published:** 2026-07-31

**Authors:** K. T. Nguyen, D. Ilki, J. Muniz, M. Grados

**Affiliations:** 1 Johns Hopkins University, Baltimore, United States; 2 Koç University Faculty of Medicine, Istanbul, Türkiye; 3 Johns Hopkins School of Medicine, Baltimore, United States

## Abstract

**Introduction:**

The Mediator complex is a 26 gene multi-subunit transcriptional regulator essential for RNA polymerase II-mediated gene expression in humans. Mediator complex mutations cause Lujan and Cornelia de Lange syndrome. More specifically, *MED13L* mutations cause neurodevelopmental disorders with structural brain abnormalities, intellectual disability and autism. Reports of psychosis in MED13L variants are not common while treatment strategies are unmapped.

**Objectives:**

A report is undertaken of the therapeutic potential of xanomeline to ameliorate psychotic symptoms in a treatment-resistant adolescent with a *MED13L* mutation.

**Methods:**

Use of xanomeline is detailed in a 15-year-old male harboring a heterozygous *de novo* missense *MED13L* variant (c.3410 A>G, p.N1137S) and a 22q11.2 duplication copy number variant.

**Results:**

A 15 year-old male with a *MED13L* deleterious point mutation presented with worsening of psychotic symptoms, and a diagnosis of schizoaffective disorder, bipolar type. Past psychiatric history was notable for prior hospitalizations in May and April 2024 for aggression, in December 2024 for aggression and suicidal ideation and in July 2025 for aggression. Prior trials include citalopram 10 mg (worsened agitation), aripiprazole 5 mg (ineffective), haloperidol 7.5 mg (ineffective), olanzapine 10 mg (ineffective), valproate 750 mg (tremors, weight gain), sertraline (worsening manic symptoms) in 2024 and Ativan 1 mg TID (disinhibition) in 2023. Given the poor response overall to various mood-stabilizing and antipsychotic medications, xanomeline at 50 mg (+ 20 mg trospium) twice daily, titrated to 100/20 mg twice daily was cross-tapered with risperidone. Over the next 2 weeks, the patient’s behavior improved, he was more social and no more aggressive episodes were evident. No significant side effects were noted from use of xanomeline.

**Conclusions:**

Xanomeline-trospium is an antipsychotic recently approved by FDA in September 2024 for use in adults with schizophrenia. Xanomeline, a muscarinic M1/M4 agonist, is given in combination with trospium to minimize GI side effects. In our patient, the drug was well tolerated and produced positive effects on psychosis and aggression in a span of 2 weeks. Unlike most antipsychotic agents, xanomeline does not exert any effect on dopamine or serotonin receptors. Most common adverse reactions include GI symptoms such as nausea, dyspepsia. Genetic variants presenting with psychotic symptoms include the 22q11 microdeletion syndrome, Prader-Willi syndrome among others. Due to the organic brain dysfunction that these syndromes entail, psychotic syndromes can follow a torpid course with limited response to current treatment options. This report considers the use of the novel low-side effect profile xanomeline-trospium for the treatment of psychosis in genetic syndromes which can be more susceptible to side effects.

**Disclosure of Interest:**

None Declared

